# Atypical Presentation of Gastrointestinal Stromal Tumor as Multiple Intra-Abdominal Cysts: A Case Report

**DOI:** 10.7759/cureus.7999

**Published:** 2020-05-06

**Authors:** Ram Prakash Gurram, Senthil Gnanasekaran, Karan Midha, Pottakkat Biju, Raja Kalayarasan

**Affiliations:** 1 Surgical Gastroenterology, Jawaharlal Institute of Postgraduate Medical Education and Research (JIPMER), Puducherry, IND

**Keywords:** gist, multiple intraabdominal cysts, clear cell, cystic gist, hydatidosis, gastrointestinal stromal tumours

## Abstract

Gastrointestinal stromal tumors (GISTs) are the most common nonepithelial solid neoplasms involving the alimentary tract. We report a case of cystic GIST with multiple cystic metastases. A 61-year-old man presented with upper abdominal pain for two months. Further evaluation revealed a large intra-abdominal cyst in the lesser sac and another cyst over the segment VII of the liver on imaging. Multiple intra-abdominal hydatidoses were suspected based on the imaging and its endemic nature in the geographical area. However, the hydatid serology was normal. In view of hemorrhagic cyst fluid, an intraoperative frozen biopsy of the cyst wall was done, which revealed features suspicious of a mesenchymal tumor. Sleeve gastrectomy with en-bloc excision of the gastric cyst, excision of the hepatic cyst, and complete excision of multiple other intra-abdominal cysts were performed considering GIST as a possibility. Histology revealed a clear cell variant of GIST. Gastric GISTs primarily presenting as multiple intra-abdominal cysts and of clear cell histological variants had never been reported in the literature. The patient was started on imatinib, and he has shown no evidence of recurrence after 12 months of follow-up.

A high index of suspicion, intraoperative frozen section, meticulous surgery, and immunohistochemistry are all crucial for the effective management of atypical cases. GIST may be considered as a part of differential diagnosis in clinical scenarios with multiple intra-abdominal cysts, especially in the equivocal setting.

## Introduction

Gastrointestinal stromal tumors (GISTs) are the most common nonepithelial solid neoplasms of the gastrointestinal tract. Although GISTs constitute less than 5% of all gastrointestinal tract tumors, they represent the most common form of sarcoma in the gastrointestinal tract [1,2]. GISTs are predominantly solid tumors with cystic changes being rare [3,4]. Intra-abdominal hydatidosis, multiple pancreatic pseudocysts, and duplication cysts are among the common differential diagnoses to be considered in the evaluation of intra-abdominal disease with multiple cysts. The present study reports a case of primary stomach GIST with intra-abdominal metastases presenting as multiple large intra-abdominal cysts.

## Case presentation

**Figure 1:**
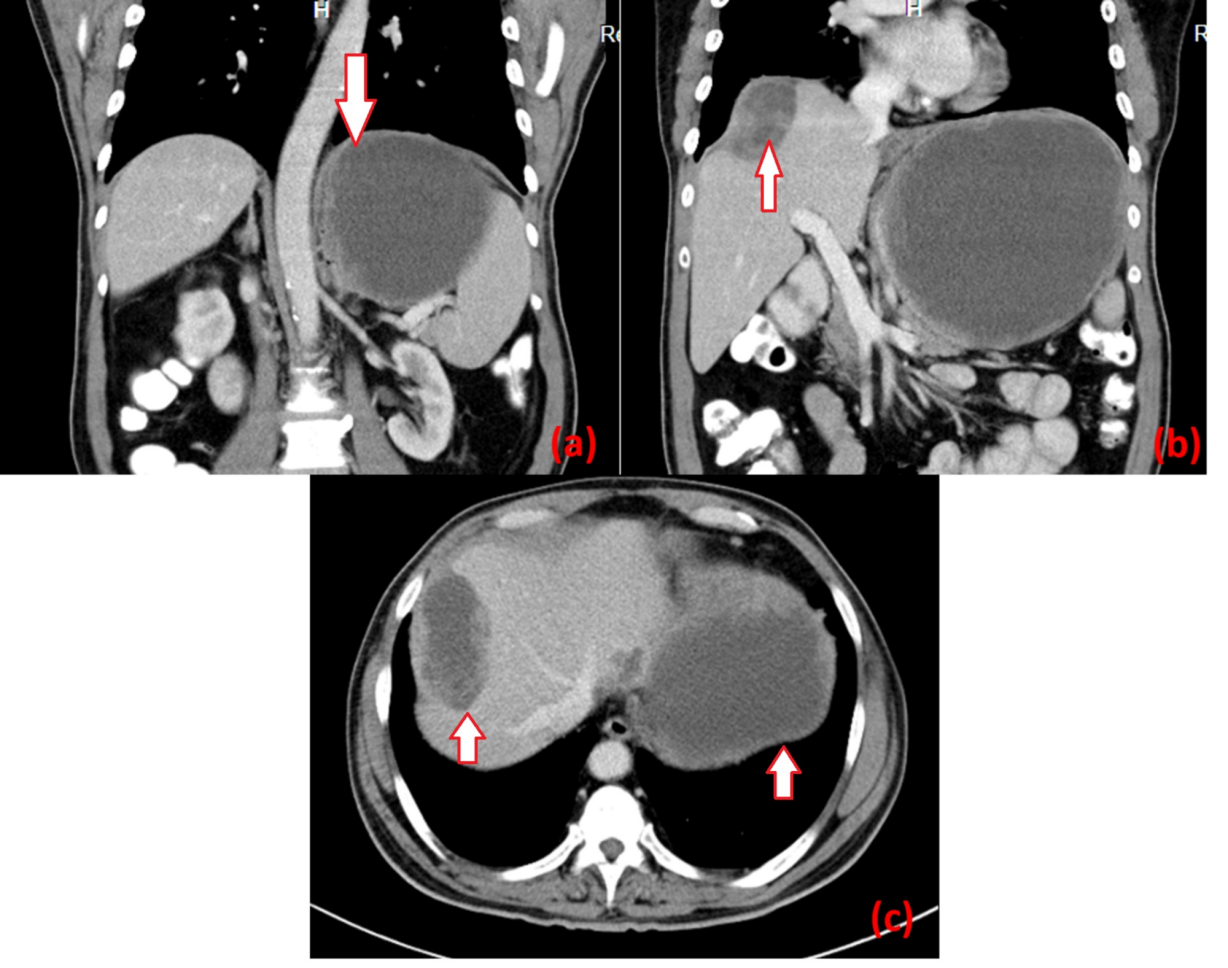
Preoperative CECT images of the abdomen (a) coronal view showing a cystic lesion in the left subphrenic region (arrow); (b) coronal view showing a cystic lesion in segment VII of the liver with thickened wall and few septations (arrow); (c) axial cut showing both liver surface lesion and left subdiaphragmatic lesions (both highlighted by arrows) CECT: contrast-enhanced computed tomography

A 61-year-old man presented with left upper quadrant abdominal pain associated with anorexia for two months. He had never had associated symptoms of gastric outlet obstruction, gastrointestinal bleed, or jaundice. A large, firm, non-tender mass was palpable in the upper abdomen, which was moving with respiration. Initially, the patient underwent ultrasonography of the abdomen, which showed Ill-defined, heteroechoic, non-septated cystic lesion without any intramural component, measuring 6.6 x 4.6 cm in segment VII of the liver, and another lesion measuring 14.3 x 12.6 cm in the left subphrenic region with similar characteristics. Contrast-enhanced CT (CECT) scan of the abdomen revealed a 17 x 14 x 14-cm, well‑defined thick-walled cystic lesion in the left subphrenic region between the left lobe of the liver and stomach. Few subtle areas of enhancement of the cyst wall with no obvious septations or mural nodules were appreciated. Another thick-walled cystic lesion measuring 7 x 4 x 5 cm was detected over the segment VII of the liver with few septations (Figure 1).

**Figure 2:**
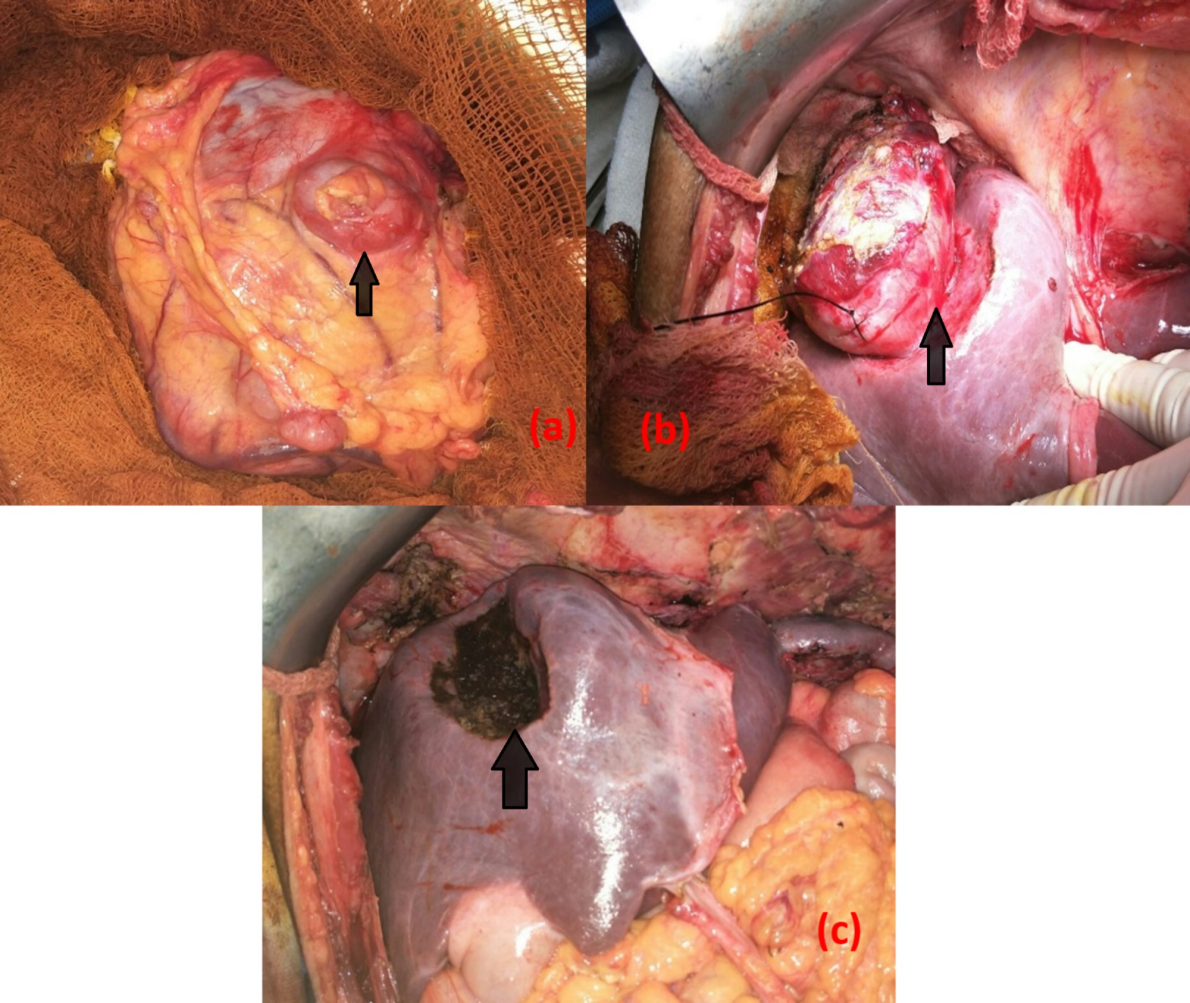
Intraoperative images (a) large cystic lesion with thickened wall and few surface nodules (arrow showing one of the nodules over lesion); (b) liver surface cystic lesion with thickened wall (arrow showing thickened wall); (c) scalloped liver surface after cyst excision (arrow showing scalloping)

No other lesion was detected in peritoneum or lung. Upper gastrointestinal endoscopy revealed extrinsic impression over fundus and body of stomach with unremarkable mucosa. Hydatid serology performed suspecting intra-abdominal hydatidosis turned out to be negative. With the suspicion of the rare possibility of a cystic tumor, degeneration tumor markers like cancer antigen 125 (CA 125), CA 19-9, and carcinoembryonic antigen (CEA) were done, but none of them showed any significant level of elevation. The patient was taken for surgery with suspicion of intra-abdominal hydatidosis after obtaining due consent for the surgical procedure. Midline laparotomy revealed a large cystic lesion of around 18 x 15 x 12 cm involving the posterolateral wall of the proximal stomach obliterating the lesser sac and adherent to the left diaphragm. Another cyst of size 7 x 4 x 4 cm was present in the right subdiaphragmatic space abutting segment VII of the liver and densely adherent to the diaphragm as well (Figure 2).

**Figure 3:**
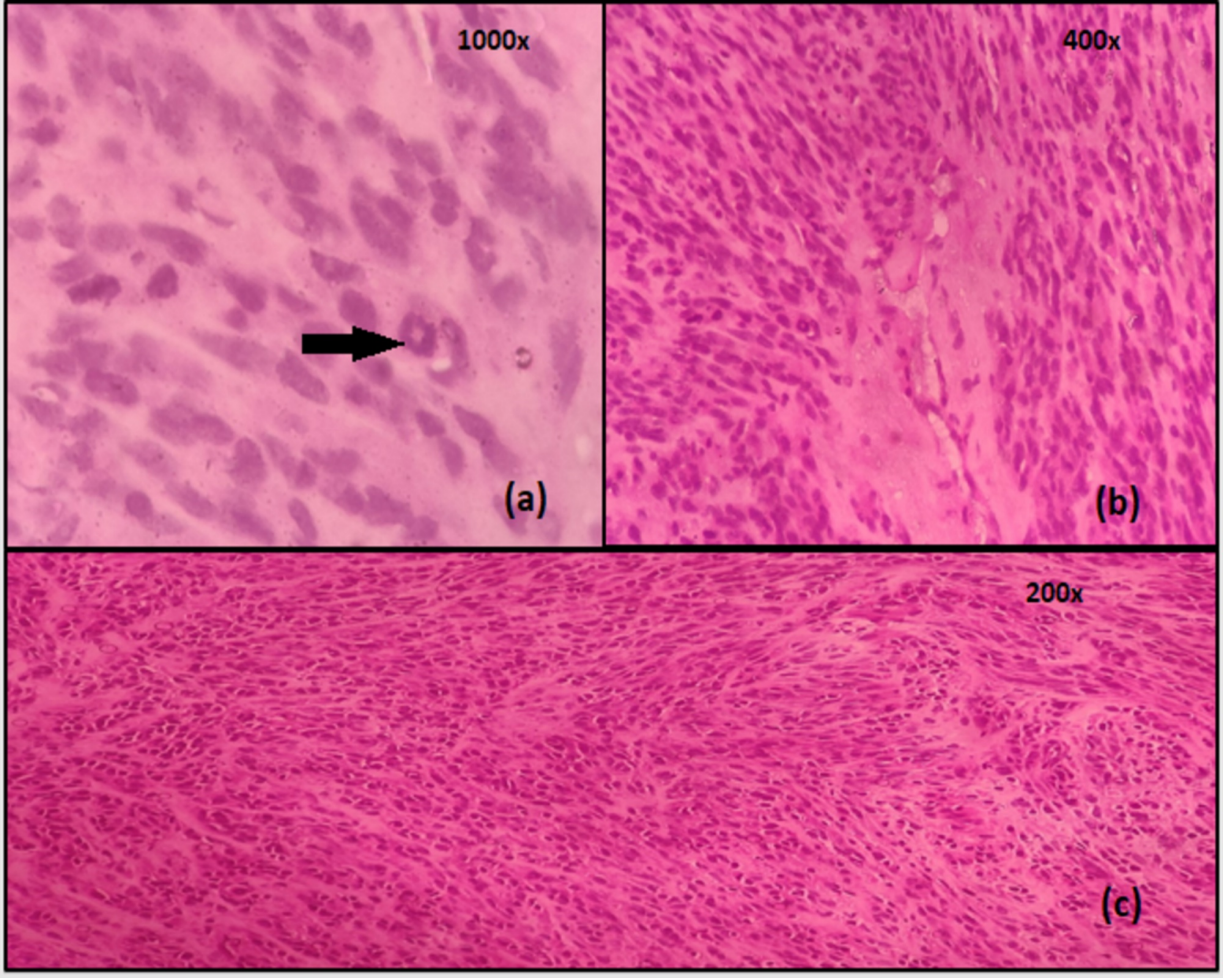
Intraoperative frozen section of the cyst wall (a) image shows ring mitoses (highlighted by the arrow); (b) image showing tumor cells exhibiting moderate nuclear pleomorphism with frequent mitoses; (c) frozen section showing spindle-shaped tumor cells arranged in short intersecting fascicles

Also, three other intra-abdominal sub centimetric cystic nodules were noted along the diaphragm and omentum. A part of the cyst wall was sent for frozen analysis since the aspirate was hemorrhagic in nature, which was not consistent with hydatidosis. The intraoperative frozen section of the cyst wall revealed sheets of spindle-shaped tumor cells arranged in short intersecting fascicles and exhibiting numerous scattered mitosis (Figure 3).

Thus, the possibility of a malignant mesenchymal tumor was considered with cystic metastases in the supracolic compartment, and the decision for definitive surgery was made. After assessing the extent of the lesion, the sleeve of the stomach was removed en bloc with a tumor followed by meticulous dissection of the rest of the cystic lesions, which had dense adhesions with surrounding structures. Scrupulous dissection of the lesion over the liver revealed scalloping of the liver surface without any obvious infiltration. The complete oncological clearance of all the cystic lesion was achieved at the end.

**Figure 4:**
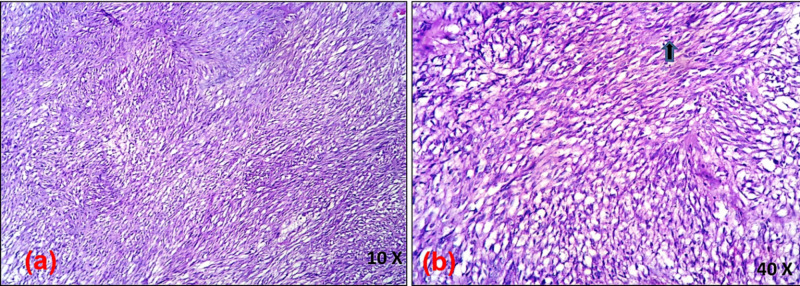
Postoperative histopathology Low (a) and high (b) magnification views showing fascicles of spindle-shaped tumor cells with clear cell morphology and an occasional mitotic figure (arrow showing mitotic figure)

Postoperatively, histopathology showed a tumor composed of sheets of cells in fascicles with clear cytoplasm with occasional areas of cells with abundant eosinophilic cytoplasm reminiscent of an epithelioid morphology (Figure 4).

**Figure 5:**
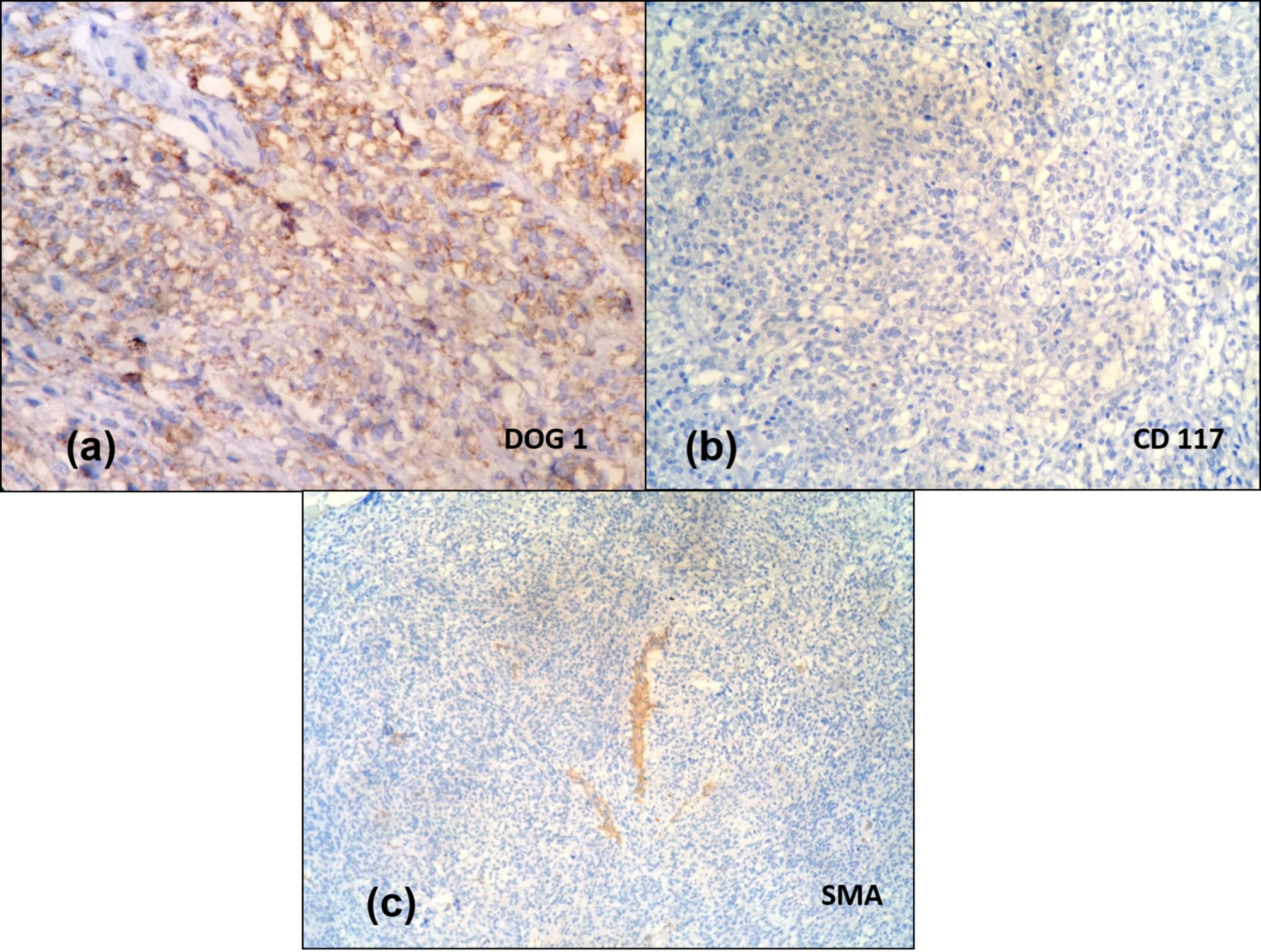
Immunohistochemical staining (a) DOG-1 shows strong membrane positivity in the tumor cells; (b) CD117-negative tumor cells; (c) SMA-negative tumor cells DOG: discovered on gastrointestinal stromal tumor; CD117: cluster of differentiation-117; SMA: smooth muscle actin

Hence, possibilities of a gastrointestinal stromal tumor, leiomyosarcoma, clear cell sarcoma-like tumor of the gastrointestinal tract, and perivascular epithelioid cell (PEComa) tumors were considered. Although the location of the tumor favored the diagnosis of GIST, clear cell morphology warranted further characterization with immunohistochemistry, which was mandatory to establish a definitive diagnosis as clear cell morphology had not been described in association with GIST previously in the literature. A battery of immunohistochemical markers that included the cluster of differentiation-117 (CD-117), DOG-1 (discovered on GIST-1 stain), CD34, SMA (smooth muscle actin), S100, CD56, Melan A, and human melanoma black-45 (HMB45) was applied to differentiate the possibilities mentioned above. DOG-1 was strongly positive, favoring GIST; however, CD117 and CD34 were negative. SMA, the specific marker for leiomyosarcoma, was negative. S100 and CD 56 negativity ruled out the diagnosis of clear cell sarcoma-like tumor of the gastrointestinal tract; similarly, the absence of staining for Melan A and HMB45 excluded PEComa as a possibility. Ultimately, a diagnosis of the gastrointestinal tumor with clear cell differentiation was made (Figure 5).

After undergoing a surgical procedure in the form of sleeve gastrectomy with en bloc excision of gastric GIST, hepatic metastatectomy, and the excision of abdominal cysts, the patient had an uneventful postoperative course. In view of the various high-risk features like large-size, multiple metastatic cystic lesions, and a mitotic rate of >5/50 high-power fields, the patient was started on postoperative imatinib therapy (400 mg once daily). As of part of our follow-up protocol, all patients with high-risk features and metastatic GIST are scheduled to undergo ultrasonography of the abdomen once every three months and CECT of the abdomen once every six months for the initial three years during adjuvant therapy. At the one-year follow-up, the patient did not have any evidence of recurrent disease.

## Discussion

Although GIST is the most common mesenchymal tumor of the gastrointestinal tract, its diagnosis can be challenging [1]. Most atypical presentations are diagnosed only on histopathological basis after surgical resection [5]. The atypical presentation of GIST in our patient, with complete cystic degeneration, is no exception for the same although the diagnosis was made intraoperatively. Multiple intrabdominal cysts in relation to liver prompted us to evaluate lesion in terms of hydatidosis owing to the endemic nature of the same in the region as well as for the other neoplasms that are known to have a cystic component. GISTs of large size, high-malignant potential GISTs, and usage of imatinib in the neoadjuvant setting are occasionally reported to have cystic components. This was hypothesized due to rapid tumor growth leading to relatively reduced vascularity in the center of mass or tumor necrosis due to chemotherapy itself, although the exact mechanism needs to be elucidated [6,7]. However, cystic degeneration is usually confined to the center of the tumor rather than the entire lesion being predominantly cystic. GIST with predominant cystic degeneration is exceedingly rare and only 11 cases have been reported in the literature to date [5]. Previously reported cases range in size from 6 cm to 37.6 cm and had mitotic counts of >5 mitoses/high-power field [6].

GIST presenting as multiple large intra-abdominal cysts apart from a primary tumor with near-complete cystic degeneration as in our case has never been reported in the literature. Clinical presentation and multiple large cystic lesions on imaging were consistent with hydatidosis although there was subtle peripheral enhancement and absence of classical septations. Failure to establish definitive diagnosis and translation of this clinical dilemma into the therapeutic domain entails the risk of the patient ending up with sub-optimal therapeutic measures. A high index of suspicion is necessary for atypical presentations, and every attempt should be made to get a precise diagnosis so as to offer the surgical therapy that adequately addresses the condition in question. Although the frozen section is not routinely used in the evaluation of GIST, the threshold for using it should be kept low in cases of any atypical findings. It can help in clinching the diagnosis with certainty in cases of clinical dilemmas like ours. Frozen findings suggestive of mesenchymal tumor aided us in further decision-making and completing an oncological resection. Histopathological examination of surgically resected specimens ascertained the diagnosis of GIST based on immunohistochemistry markers and led to further insights of its epithelioid variant with clear cell subtype. Corresponding to the peculiar clinical presentation, histopathology revealed an equally distinctive entity. Clear cell morphology corresponding to a multicystic variant had not been reported in scientific literature to date. As the biological potential and morphological spectrum depend relatively on various subtypes of GIST, its identification is of clinical importance [2]. The clear cell morphology may be an indicator of high-grade GIST with metastatic potential. 

Knowledge about the condition and a high index of suspicion are critical in the diagnosis of GIST with atypical presentation. We suggest that physicians consider the intraoperative frozen section in such a scenario for making further decisions. GIST should also be considered as a part of differential diagnosis in the evaluation of multicystic lesions of uncertain etiology as shown in this case. When the diagnosis is established, oncologically complete excision should be done whenever feasible.

## Conclusions

Our evaluation of a multicystic abdominal disease of unknown etiology led us to the diagnosis of a clear cell variant of GIST. The initially suspected diagnosis of hydatidosis was changed intraoperatively with valuable inputs from the frozen section. With a diagnosis of GIST in mind, the patient underwent oncological R0 resection. Postoperative histopathology established the diagnosis of clear cell variant of GIST after immunohistochemistry. Ultimately, the patient remained recurrence-free after 12 months of follow-up on adjuvant imatinib therapy. A high index of suspicion, intraoperative frozen section, R0 resection, and immunohistochemistry all played pivotal roles in helping us address this clinical dilemma.
